# Japanese Encephalitis DNA Vaccines with Epitope Modification Reduce the Induction of Cross-Reactive Antibodies against Dengue Virus and Antibody-Dependent Enhancement of Dengue Virus Infection

**DOI:** 10.3390/vaccines10091411

**Published:** 2022-08-28

**Authors:** Tomohiro Kotaki, Yurie Nagai, Atsushi Yamanaka, Eiji Konishi, Masanori Kameoka

**Affiliations:** 1 Department of Public Health, Kobe University Graduate School of Health Sciences, Kobe 654-0142, Japan; 2 Department of Virology, Research Institute for Microbial Diseases, Osaka University, Osaka 565-0871, Japan; 3 BIKEN Endowed Department of Dengue Vaccine Development, Faculty of Tropical Medicine, Mahidol University, Bangkok 10400, Thailand; 4 Mahidol-Osaka Center for Infectious Diseases, Faculty of Tropical Medicine, Mahidol University, Bangkok 10400, Thailand

**Keywords:** Japanese encephalitis virus, dengue virus, vaccine, antibody-dependent enhancement

## Abstract

Infection with viruses belonging to the genus *Flavivirus*, such as Japanese encephalitis virus (JEV) and dengue virus (DENV), is a worldwide health problem. Vaccines against JEV and DENV are currently available. However, the dengue vaccine possibly increases the risk of severe dengue due to antibody-dependent enhancement (ADE). Moreover, the Japanese encephalitis (JE) vaccine reportedly induces cross-reactive ADE-prone antibodies against DENV, potentially leading to symptomatic dengue. Therefore, it is necessary to eliminate the risk of ADE through vaccination. In this study, we attempted to develop a JE vaccine that does not induce ADE of DENV infection using an epitope modification strategy. We found that an ADE-prone monoclonal antibody cross-reactive to DENV and JEV recognizes the 106th amino acid residue of the E protein of JEV (E-106). The JE DNA vaccine with a mutation at E-106 (E-106 vaccine) induced comparable neutralizing antibody titers against JEV to those induced by the wild-type JE DNA vaccine. Meanwhile, the E-106 vaccine induced 64-fold less cross-reactive ADE-prone antibodies against DENV. The mutation did not compromise the protective efficacy of the vaccine in the lethal JEV challenge experiment. Altogether, the modification of a single amino acid residue identified in this study helped in the development of an ADE-free JE vaccine.

## 1. Introduction

The genus *Flavivirus* of the family *Flaviviridae* includes several clinically important mosquito-borne viruses, such as the Japanese encephalitis virus (JEV) and dengue virus (DENV). JEV causes 50,000–175,000 cases of Japanese encephalitis (JE) annually, primarily in Southeast Asian countries [1]. The case fatality rate can reach as high as 30% [2]. Moreover, it has been reported that 20–30% of individuals who survive JE develop neurological sequelae [2]. A vaccine against JE was approved in Japan in 1954 [3]. The efficacy of the JE vaccine was >90%, and its safety was also well confirmed [2,4]. Therefore, 24 countries have included JE vaccines in their routine immunization program [5]. This measure has reduced the number of patients with JE in these countries [2,6,7].

Four serotypes of DENV (DENV-1 to DENV-4) have been reported to have caused 100 million cases of dengue and severe dengue (also known as dengue hemorrhagic fever) in tropical and subtropical countries [8,9]. Although the case fatality rate is <1%, 21,000 fatal cases are reported every year [8,9]. Vaccination against dengue has been recently approved in 20 dengue-endemic countries [10]. However, its efficacy is not high among individuals who have not experienced a previous DENV infection (i.e., seronegative individuals) [11]. Moreover, this vaccination may increase the risk of severe dengue symptoms, possibly through the antibody-dependent enhancement (ADE) of the viral infection [11,12]. ADE is a phenomenon in which non-neutralizing or weak-neutralizing antibodies enhance the viral infection of Fc receptor-bearing cells [13]. Additionally, it is well known that patients who have experienced a previous DENV infection tend to develop severe dengue during secondary infection with heterotypic DENV [14]. Therefore, pre-existing anti-DENV antibodies induced by either natural infection or vaccination may facilitate subsequent DENV infections.

The viruses belonging to the genus *Flavivirus* (hereafter flaviviruses) have a positive-strand RNA genome that encodes three structural proteins (capsid [C], premembrane [prM], and envelope [E]) and seven nonstructural proteins (NS1, NS2A, NS2B, NS3, NS4A, NS4B, and NS5) [15]. The E protein is located on the surface of the virus and is the target of vaccine antigens for flavivirus infections. Additionally, the E protein has a fusion loop (FL) region responsible for the membrane fusion of the virus [16]. The FL region is highly conserved, particularly among mosquito-borne flaviviruses. Therefore, antibodies that target the FL region are usually highly cross-reactive among these flaviviruses [17,18]. Furthermore, flavivirus infection induces cross-reactive antibodies against other flaviviruses [19,20]. False-positive results obtained in the serological diagnosis of flavivirus infections are attributed to the high cross-reactivity of these antibodies [21]. Importantly, antibodies that target the FL region are generally weakly neutralizing [22]. These antibodies are considered to be involved in ADE due to their high cross-reactivity and weak neutralization characteristics.

JE vaccination has been reported to induce antibodies that are cross-reactive to DENV, which causes the ADE of DENV infections in vitro [23]. Approximately 45% of individuals who receive JE vaccines have antibodies causing the ADE of the DENV infection. A study in Thailand reported a higher number of symptomatic dengue cases in JE-vaccinated individuals than in nonvaccinated individuals [24]. Furthermore, a Japanese traveler who received a JE vaccine was reported to develop severe dengue during the primary DENV infection [25]. Therefore, as JE vaccination is implemented in the routine vaccination programs of several countries, numerous individuals may be vulnerable to DENV infection. Although the impact of JE vaccination on subsequent DENV infection remains controversial [4], it is necessary to eliminate the possibility of a high risk of symptomatic DENV infection.

Epitope-modified flavivirus antigens have been receiving increasing attention. Regarding diagnosis, abrogated cross-reactive epitope antigens have been found to improve the accuracy of the serological diagnosis of mosquito-borne flavivirus infections, including those caused by JEV [26,27], West Nile virus [28], and DENV [29]. Furthermore, as a vaccine, FL-modified antigens have demonstrated a reduced induction of ADE-prone antibodies in DENV [30,31,32] and Zika virus vaccine models [33]. However, there are no reports on epitope-modified JE vaccines.

We have previously established an anti-DENV mouse monoclonal antibody, D1-V-3H12 (hereafter 3H12), that exhibits strong ADE activity but no neutralizing activity [34]. The deduced epitope of 3H12 contains the 107th amino acid of the E protein of DENV-1 [32]. Dengue DNA vaccines containing L107F exhibit a reduced induction of ADE activity in mice [32]. As 3H12 is cross-reactive to JEV, we have hypothesized that a 3H12-like antibody is involved in the ADE of DENV infections in JE vaccination. Consequently, the abrogation of the 3H12 epitope in the JE vaccine may reduce the induction of such ADE-prone antibodies. In this study, we aimed to identify a 3H12 epitope on JEV and generate epitope-modified JE DNA vaccines that can reduce the induction of cross-reactive ADE-prone antibodies against DENV.

## 2. Materials and Methods

### 2.1. Ethics

The Animal Experiment Committee of Kobe University (Ethics Committee Approval Number: P180504) approved the animal experiments. Trained laboratory personnel used isoflurane inhalation to anesthetize mice during DNA immunization, viral inoculation, and cervical dislocation euthanasia. Following the virus challenge, mice showing a decrease of 20% in their initial body weight were humanely euthanized. All experiments were conducted according to the ARRIVE guidelines (https://arriveguidelines.org, Accessed on 26 August 2022) and other relevant guidelines and regulations.

### 2.2. Cell Lines

HEK293T cells were maintained in Dulbecco’s Modified Eagle Medium supplemented with 10% fetal bovine serum (FBS). Vero cells were cultured in Eagle’s Minimum Essential Medium (MEM) supplemented with 10% FBS and 60 μg/mL kanamycin. C6/36 cells were cultured in Eagle’s MEM supplemented with 10% FBS, nonessential amino acids, and 60 μg/mL kanamycin. K562 erythroleukemia cells were cultivated in RPMI 1640 medium supplemented with 10% FBS, 100 units/mL penicillin, and 100 μg/mL streptomycin.

### 2.3. Viruses and Antibodies

The prototype viruses used in this study included the JEV Nakayama, DENV-1 Mochizuki, DENV-2 NGC, DENV-3 H87, and DENV-4 H241 strains [34,35]. The JEV vaccine strains Beijing-1 and P3 were also used [35]. Viruses were propagated in C6/36 cells for 2–5 days and stored at −80 °C until use.

A mouse monoclonal antibody named 3H12 was used for its epitope mapping [34]. D1-4G2 (anti-E protein, cross-reactive to the Flavivirus group; American Type Culture Collection, Manassas, VA, USA) was used to detect virus-infected cells. Furthermore, the sera of mice and rabbits immunized with inactivated JEV for more than 3 times were used as anti-JEV polyclonal antibodies [36].

### 2.4. Virus Titration and Immunostaining

Infective viral titers were determined as described previously [37]. Briefly, Vero cells were seeded in a 96-well plate (2 × 10^4^ cells/well). The next day, serially diluted virus culture supernatants were inoculated, followed by overnight incubation at 37 °C. The cells were fixed in 4% paraformaldehyde–phosphate buffer saline (PBS). The cells were then serially incubated with D1-4G2, biotinylated anti-mouse immunoglobulin G (IgG), a VECTASTAIN Elite ABC kit (Vector Laboratories, Burlingame, CA, USA), and a VIP peroxidase substrate kit (Vector Laboratories, Burlingame, CA, USA). Viral foci were manually counted and the titer was expressed as a focus-forming unit (FFU).

### 2.5. Escape Mutant Generation and Sequence Analysis

Vero cells seeded in a 6-well plate (3 × 10^5^ cells/well) were infected with the JEV Nakayama strain at a multiplicity of infection (MOI) of 0.1. After 1 h of incubation at 37 °C, the cells were washed thrice with PBS and then cultured with the Vero cell culture medium containing 3H12 at a concentration of 16.0 or 8.0 μg/mL. Culture supernatants were transferred to new cells after 4 days of incubation. This procedure was repeated 5 times. Moreover, the passage control virus, which was passaged in the absence of an antibody, was generated.

Viral RNA was extracted from the culture supernatant of the cells infected with escape mutant using TRIzol reagent (Invitrogen, Carlsbad, CA, USA). Reverse transcription polymerase chain reaction (RT-PCR) was performed using the Superscript RT-PCR system (Invitrogen, Carlsbad, CA, USA) and Ex Taq (Takara, Shiga, Japan) to amplify the E gene. Primer information is presented in Supplementary Table S1. The E gene sequences between the escape mutant and passage control virus were compared using Genetyx ver. 10 (Genetyx, Tokyo, Japan).

### 2.6. Plasmid Preparation

The pcDNA3.1 plasmids encoding the prM–E genes of JEV (Nakayama, Beijing-1, and P3 strains) or DENV-2 were constructed as previously described [38]. Mutations (G106V and/or L107F) were then introduced into the plasmids using a site-directed mutagenesis kit (Toyobo, Osaka, Japan). Primer information is shown in Supplementary Table S1. These plasmids were used as DNA vaccines in this study.

### 2.7. Preparation of Virus-like Particles and Their Binding to 3H12

HEK293T cells were seeded in a 24-well plate (6 × 10^4^ cells/well). The next day, the HEK293T cell monolayer was transfected with 600 ng of DNA plasmids using the Fugene HD transfection reagent (Promega, Madison, WI, USA). The culture supernatant containing virus-like particles (VLPs) was harvested 3 days after transfection. The harvested VLPs were subjected to an enzyme-linked immunosorbent assay (ELISA) to determine their amount and reactivity to 3H12.

### 2.8. Quantification and Antigenic Analysis of VLPs Using ELISA

Quantification and antigenic analysis using ELISA was performed according to a previous report [39]. Rabbit serum immunized with the JEV Nakayama strain was coated onto Nunc Maxisorp ELISA plates (Thermo Fisher Scientific, Waltham, MA, USA) to quantify VLPs. Next, the VLPs from plasmid-transfected 293T cells, anti-JEV mouse polyclonal antibody, alkaline phosphatase (AP)-conjugated anti-mouse IgG (Jackson ImmunoResearch Laboratories, West Grove, PA, USA), and p-nitrophenyl phosphate (PNPP) (Nacalai Tesque, Kyoto, Japan) were serially incubated, and the absorbance was measured at 415 nm. The ELISA diluent comprised PBS containing 1% bovine serum albumin and 0.05% Tween 20. The number of VLPs was expressed as the optical density (OD) of mutant VLP relative to that of wild-type VLP (OD of mutant VLP/OD of wild-type VLP).

For antigenic analysis, the same ELISA procedure was followed, but using 3H12 as the detector antibody. Additionally, the reactivity of the VLPs was expressed as the OD of mutant VLP relative to that of wild-type VLP (OD of mutant VLP/OD of wild-type VLP).

### 2.9. DNA Vaccine Immunization

Six-week-old male BALB/c mice were immunized twice or thrice with 100 μg of DNA vaccine intratibially using a NEPA21 electroporator (Nepa Gene, Chiba, Japan) at 2-week intervals, as described in a previous report [32]. Six or seven mice were used in each group. Blood samples were collected 1 week after the last immunization. Sera samples isolated from the blood samples were heat-inactivated at 56 °C for 30 min and then subjected to antibody analyses, including an ELISA, neutralization test, and ADE assay.

### 2.10. Semi-Quantification of Anti-JEV Antibodies Induced by DNA Vaccines

Rabbit polyclonal antibody was coated onto the plate as described earlier. Next, the JEV Nakayama strain (1.0 × 10^5^ FFU/well), serially diluted immunized mouse serum, AP-conjugated anti-mouse IgG, and PNPP were serially incubated, followed by the measurement of absorbance.

IgG1 and IgG2a titers were measured as previously described [40]. Virus coating was performed followed by the addition of diluted mouse serum as described earlier. AP-conjugated anti-mouse IgG1 or IgG2a was used as the secondary antibody, and color development was performed. The IgG1 and IgG2a titers were expressed as the maximum dilution yielding a 2-times higher value than the negative-control average.

### 2.11. Neutralization Test

Neutralization tests were performed as described previously [37]. Briefly, Vero cells were seeded in a 96-well plate (2 × 10^4^ cells/well). The next day, 100 FFU of virus and serially diluted serum were mixed and incubated at 37 °C for 1 h, followed by inoculation onto the cells. The following day, the cells were fixed and immunostained. The neutralizing antibody titer was expressed as the maximum serum dilution yielding a 50% reduction in the focus number (NT_50_).

### 2.12. ADE Assay

ADE activity was measured using semi-adherent K562 cells, as described previously [41]. Briefly, serially diluted sera samples were mixed with 100 FFU of DENV; these samples were incubated at 37 °C for 2 h in a poly-l-lysine-coated plate in the presence or absence of rabbit complement at a final concentration of 5% (Cedarlane Laboratories, Burlington, Canada) [32]. Next, 1 × 10^5^ K562 cells were added to the mixture and incubated for 2 days. After immunostaining, viral-infected cells were manually counted. The baseline of the infected cells (without antibody) was log_10_ 2.0 (100 FFU). The infected cell number, which is more than log_10_ 2.5 (i.e., approximately three-fold enhancement from the baseline infected cells), was considered to be ADE. The ADE titer was defined as the maximum serum dilution factor exceeding the ADE threshold.

### 2.13. In Vivo Virus Challenge Experiment

A serially diluted JEV P3 strain was inoculated intraperitoneally into six or seven 6-week-old BALB/c mice to determine the 50% lethal dose (LD_50_). The mice were monitored for 20 days. The estimated LD_50_ was 1.4 × 10^5^ FFU (Supplementary Figure S1).

Six or seven 4-week-old BALB/c mice in each group were immunized with the JE DNA vaccine (Beijing-1 strain) as described earlier. At 2 weeks after immunization, approximately 70 LD_50_ (1 × 10^7^ FFU) of the JEV P3 strain were inoculated i.p. under anesthesia. The mice were monitored for 20 days. Mice were humanely euthanized if they showed apparent symptoms or a decrease of >20% in their initial body weight.

### 2.14. Statistical Analysis

All calculations were performed using GraphPad Prism 8 (GraphPad Software Inc., San Diego, CA, USA). All experiments were repeated at least twice independently. Therefore, all figures present the average and standard deviation values of at least two independent experiments.

## 3. Results

### 3.1. Generation of Escape Mutants Using JEV and an ADE-Prone Mouse Monoclonal Antibody

**Figure 1:**
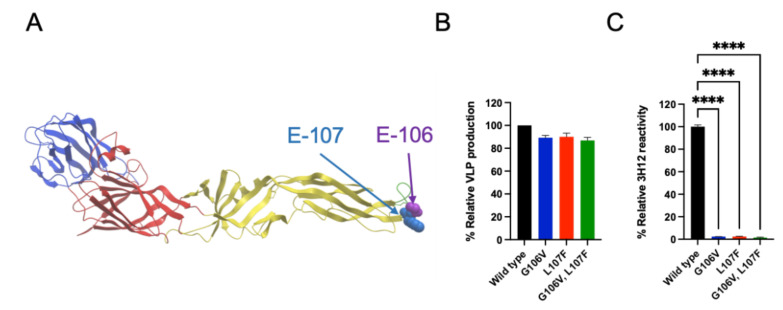
Construction of mutant JE DNA vaccines. (**A**) Location of the mutation introduced into the escape mutant using JEV and 3H12. The E protein ribbon diagram is based on Protein Data Bank (PDB) accession No. 3P54. Domains I, II, and III are denoted in red, yellow, and blue, respectively. The fusion loop (FL) region is indicated in green. The 106th and 107th amino acid residues of the E protein are highlighted as spheres. (**B**) Relative virus-like particle (VLP) production from cells transfected with JE DNA vaccines. VLPs were produced in 293T cells transfected with DNA vaccines, and quantification was conducted using ELISA. The VLP production was expressed as the optical density (OD) of mutated VLP relative to that of wild-type (OD of mutated JE vaccine/OD of wild-type vaccine). (**C**) The reactivity of 3H12 to FL-mutated JEV VLPs. The VLPs were produced in cells transfected with mutated DNA vaccines. The reactivity of 3H12 was expressed as the OD of mutated VLP relative to that of wild-type VLP (OD of mutated VLP/OD of wild-type VLP). A one-way analysis of variance with Dunnett’s post hoc test was performed to analyze the statistical significance of the comparison between the wild-type and mutant vaccines. *p* values of <0.05 were considered statistically significant. Statistically significant values (**** *p* < 0.0001) are represented in the graph.

The deduced epitope of the ADE-prone antibody, 3H12, contains the 87th and 107th amino acid residues of the E protein of DENV-1 [32]. We further conducted epitope mapping by generating escape mutants using the JEV Nakayama strain. After passaging for 5 times in the presence of 3H12, two types of escape mutants were generated. The escape mutants acquired the G106V or L107F mutation at the E protein. Both mutations were located within the FL region (Figure 1A). L107F was identical to the mutation observed in DENV-1 that escaped from 3H12 [32]. Interestingly, G106V was first identified in this study. These two residues are speculated to be a part of the enhancing antibody epitope shared by DENV and JEV.

### 3.2. Construction of JE DNA Vaccines with FL Mutations

The G106 and L107 in the JEV E protein are potentially involved in the induction of cross-reactive ADE-prone antibodies. To determine the antigenicity and immunogenicity of this epitope, DNA plasmids expressing prM–E proteins from the JEV Nakayama strain with G106V and/or L107F mutations were generated (DNA vaccines). We selected the DNA vaccine immunization model owing to the simplicity of its procedures and to have consistency with our previous work [32]. Although DNA immunization could differ from the live virus or protein immunization in terms of induced immunity, it is an appropriate first approach for determining immunogenicity.

When these plasmids were transfected into 293T cells, the mutated plasmids produced similar levels of VLPs to the wild-type plasmid in the supernatant (Figure 1B), indicating that these mutations do not impair VLP production. However, the ELISA results showed that the mutated VLPs completely lost reactivity to 3H12 (Figure 1C), validating that these two residues are a part of the 3H12 epitope.

### 3.3. Induction of Antibodies against JEV

**Figure 2:**
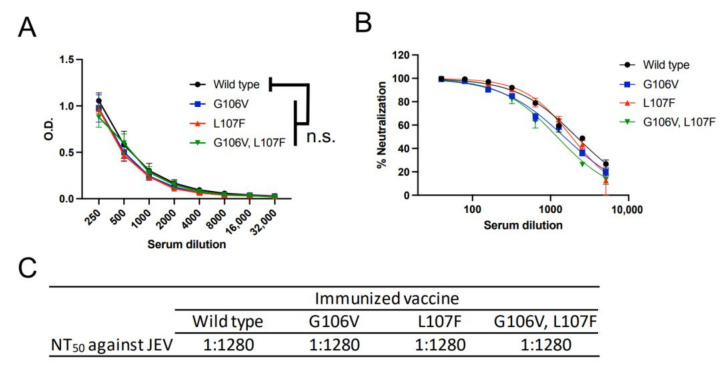
Evaluation of anti-JEV antibodies induced by JE vaccines. (**A**) Comparison of antibody induction using ELISA. JEV was coated onto the ELISA plate. Serially diluted serum samples were used as detector antibodies. A two-way analysis of variance was performed to analyze the statistical significance of the comparison between wild-type and mutant vaccines. *p* values of <0.05 were considered statistically significant. n.s., not significant. (**B**) Comparison of neutralizing activity against JEV. (**C**) NT_50_ values against JEV. NT_50_ is calculated as a maximum serum dilution yielding >50% neutralization.

The JE DNA vaccines were administered to BALB/c mice for analyzing immunogenicity. Overall, 100 μg of DNA vaccine—an adequate dose for inducing robust immunity—was administered twice at 2-week intervals through the intratibial route [32]. There was no significant difference in the induction of antibodies against JEV between the wild-type and mutated DNA vaccines (Figure 2A). The difference in the levels of neutralizing antibody titers to JEV was within two-fold (Figure 2B,C). Altogether, the mutant DNA vaccines induced comparable levels of anti-JEV antibodies to those induced by the wild-type DNA vaccine.

### 3.4. Induction of Cross-Reactive Antibodies against DENV

**Figure 3:**
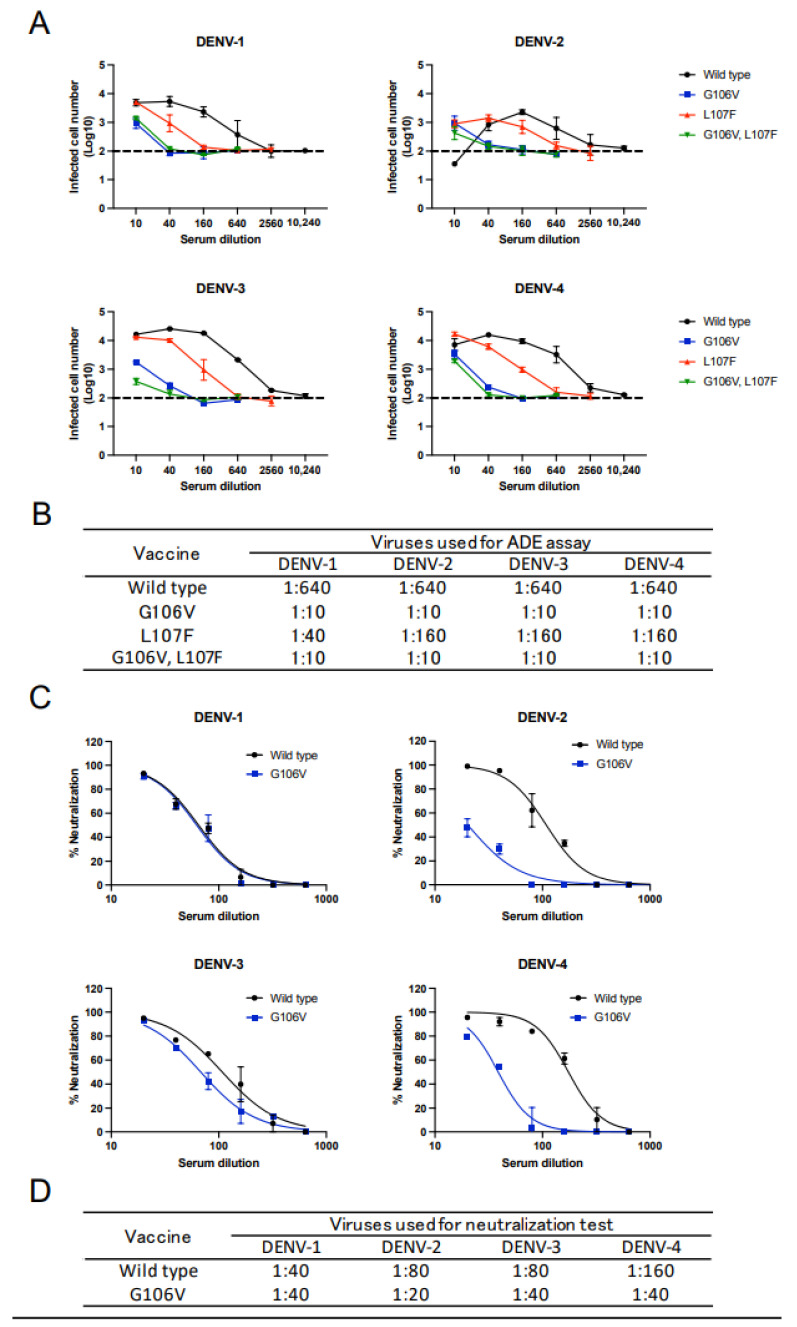
Evaluation of anti-DENV antibodies induced by JE vaccines. (**A**) ADE activity against four serotypes of DENV. Dotted lines indicate the baseline of infected cells in control (100 infected cells; log_10_ 2.0). Complement was not added to this assay. (**B**) ADE titer. The ADE titer is a maximum serum dilution factor exceeding the ADE threshold (log_10_ 2.5). (**C**) Neutralizing activity against four serotypes of DENV. (**D**) NT_50_ values against DENV. NT_50_ is calculated as a maximum serum dilution yielding >50% neutralization.

We measured ADE activity against all four serotypes of DENV using K562 cells [41] (Figure 3A,B). The ADE titer determined by this in vitro ADE assay is positively correlated with the viremia level in patients with dengue [42]. Therefore, this assay may help predict the level of viremia in subsequent DENV infections, which are associated with symptomatic or severe dengue [42,43]. The sera samples immunized with the wild-type JE DNA vaccine showed ADE activity even at a 640-fold dilution, indicating the induction of cross-reactive ADE-prone antibodies against DENVs. However, the vaccine with the L107F mutation showed ADE activity at 1:40 (DENV-1) or 1:160 (DENV-2, DENV-3, and DENV-4) dilution, indicating a reduction in ADE titers by 4–16 times. Furthermore, the vaccine with G106V mutation showed ADE activity only at 1:10 dilution in all serotypes of DENV, indicating a reduction in ADE titers by 64 times. The vaccine with the G106V/L107F mutation showed a similar pattern of ADE activity to that of the G106V-only vaccine. These data revealed that the vaccine with the G106V mutation suppressed the induction of cross-reactive ADE-prone antibodies to a higher degree than that with the L107F mutation. Additionally, G106V alone was sufficient to suppress the induction of ADE-prone antibodies.

At 1:10 serum dilution, only the combination of the wild-type JE vaccine and DENV-2 showed a tendency toward neutralization (below the baseline). It was reported that the DENV-2 NGC strain was efficiently neutralized by antibodies targeting the FL region compared with other serotypes of DENV in our ADE assay system [37]. This result is consistent with the observation in the present study: DENV-2 was efficiently neutralized by the wild-type JE vaccine, which induces antibodies against the FL region, but not by other mutated vaccines, which show a reduced induction of FL antibodies.

Furthermore, we evaluated the cross-neutralization of DENVs (Figure 3C,D). The representative mutation, G106V, reduced the neutralizing titers against DENV-2, DENV-3, and DENV-4. Neutralization of DENV-1 was not affected. These data suggested that the mutations in the FL region, especially G106V, reduced the induction of not only ADE-prone antibodies but also neutralizing antibodies against DENV. Overall, cross-reactive antibodies against DENV were less strongly induced by the JE DNA vaccine with the G106V mutation.

**Figure 4:**
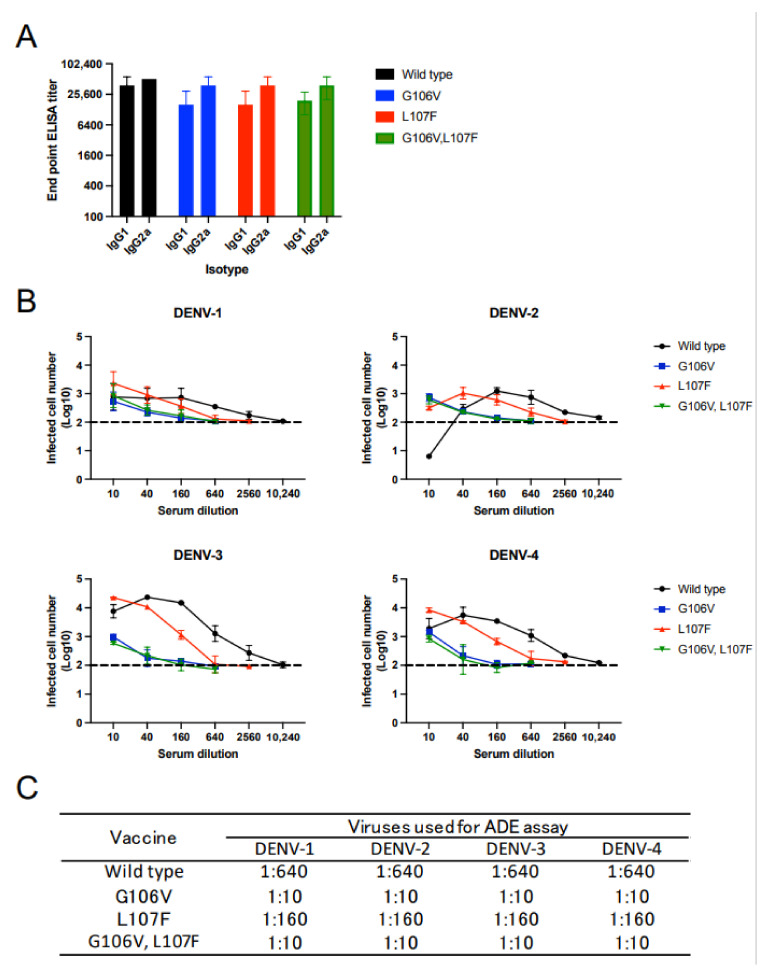
Analysis of Th1/Th2 balance induced by the vaccines. (**A**) IgG1 and IgG2a levels induced by JE DNA vaccines. JEV was coated onto the ELISA plate. Serially diluted serum was used as the detector antibody. Additionally, AP-conjugated antibodies against IgG1 or IgG2a were added for color development. The endpoint titer is expressed as the maximum dilution yielding a two-times higher value than the negative-control average. (**B**) ADE activity in the presence of complement. Dotted lines indicate the baseline of the control-infected cells (100 infected cells; log_10_ 2.0). Complement was added to investigate the complement-dependent neutralization activity. (**C**) ADE titer. The ADE titer is a maximum serum dilution factor exceeding the ADE threshold (log_10_ 2.5).

The balance of Th1/Th2 immunity affects the subtype of the induced IgG, which in turn affects ADE activity [34,40]. To further analyze the mechanism of the reduction in ADE activity, we investigated whether the mutations altered the Th1/Th2 immunity pattern. The induction patterns of IgG1 and IgG2a, which reflect the Th1/Th2 immunity pattern, were measured using ELISA. The pattern of IgG subtype induction was similar between the wild-type and mutated vaccines (Figure 4A). These data revealed that the reduction in ADE activity was due to the reduced induction of antibodies targeting the FL region and not due to the alteration of the Th1/Th2 immunity pattern or the induced IgG subtype.

The difference in the ADE activity by IgG subtype was further clarified in the presence of complement because C1q-binding ability (i.e., complement-dependent neutralization ability) is different among IgG subtypes. For example, murine IgG2a is capable of complement binding and subsequent complement-dependent neutralization, but IgG1 is not. Thus, complement addition suppresses ADE activity if IgG2a-biased (i.e., Th1-biased) immunity is induced in a polyclonal setting in mice [44,45]. Therefore, we measured the ADE activity again in the presence of complement. However, the result was similar to that without complement (Figure 3A and Figure 4B,C), corroborating that the Th1/Th2 immunity pattern was not altered.

### 3.5. Introduction of G106V Mutation into Other JEV Strains

**Figure 5:**
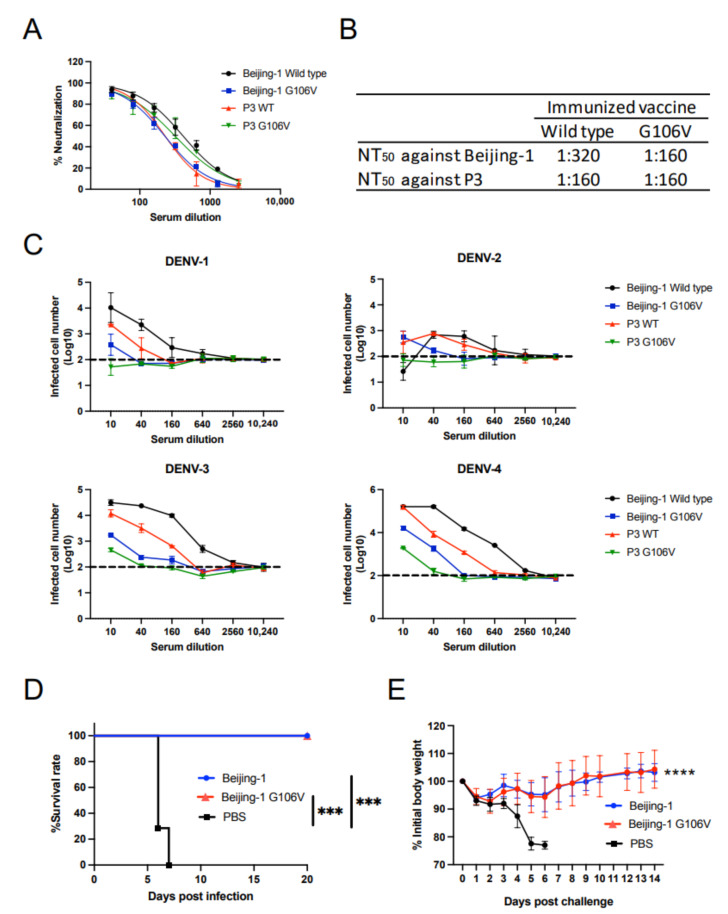
Effect of G106V mutation on JEV vaccine strains. (**A**) Neutralizing activity against JEV. (**B**) NT_50_ values against JEV. NT_50_ is calculated as a maximum serum dilution yielding >50% neutralization. (**C**) ADE activity against four serotypes of DENV. Dotted lines indicate the baseline of infected cells in control (100 infected cells; log_10_ 2.0). Complement was not added to this assay. (**D**) Survival of vaccine-administered mice after JEV challenge. A group of six BALB/c mice was infected with 70 LD_50_ of the JEV P3 strain. Statistical significance was analyzed using a log-rank test. *** *p* < 0.001 for the comparison between vaccinated and unvaccinated mice. (**E**) Body weight change in mice after JEV challenge. Statistical significance was analyzed using repeated measures two-way analysis of variance. **** *p* < 0.0001 for the comparison between vaccinated and unvaccinated mice.

To confirm whether FL mutation is applicable to other JEV vaccine strains, including Beijing-1 and P3, we constructed DNA vaccines containing the prM–E of these strains. G106V was introduced into the DNA vaccines because G106V alone was sufficient to reduce the induction of cross-reactive antibodies against DENV (Figure 3A). As anticipated, the neutralizing titers against JEV induced by mutated vaccines were comparable to those induced by wild-type vaccines (Figure 5A,B). Additionally, the ADE activity from DENV-1 to DENV-4 was reduced in the mutated vaccine group (Figure 5C). These results indicate that the mutation is applicable irrespective of the JEV strains.

To evaluate whether the protective efficacy of the mutated JE vaccine was compromised, an in vivo challenge experiment was conducted. The DNA vaccines containing Beijing-1 prM–E were used because the Beijing-1 strain is commonly used for JE vaccination [46]. The P3 strain demonstrated high virulence and was used as the challenge virus. Two weeks after the first immunization, 70 LD_50_ of the P3 strain was infected via the i.p. route. The mice that lost 20% of their initial body weight showed apparent symptoms such as paralysis. The PBS-immunized mice died within 6 days after infection. However, even 20 days after infection, all mice immunized with wild-type or G106V vaccines survived (Figure 5D). There was no difference in the body weight change between the wild-type and G106V vaccine groups (Figure 5E). These results indicate that the G106V mutation does not compromise the protective efficacy of the vaccine.

### 3.6. Effects of G106V Mutation on DENV

**Figure 6:**
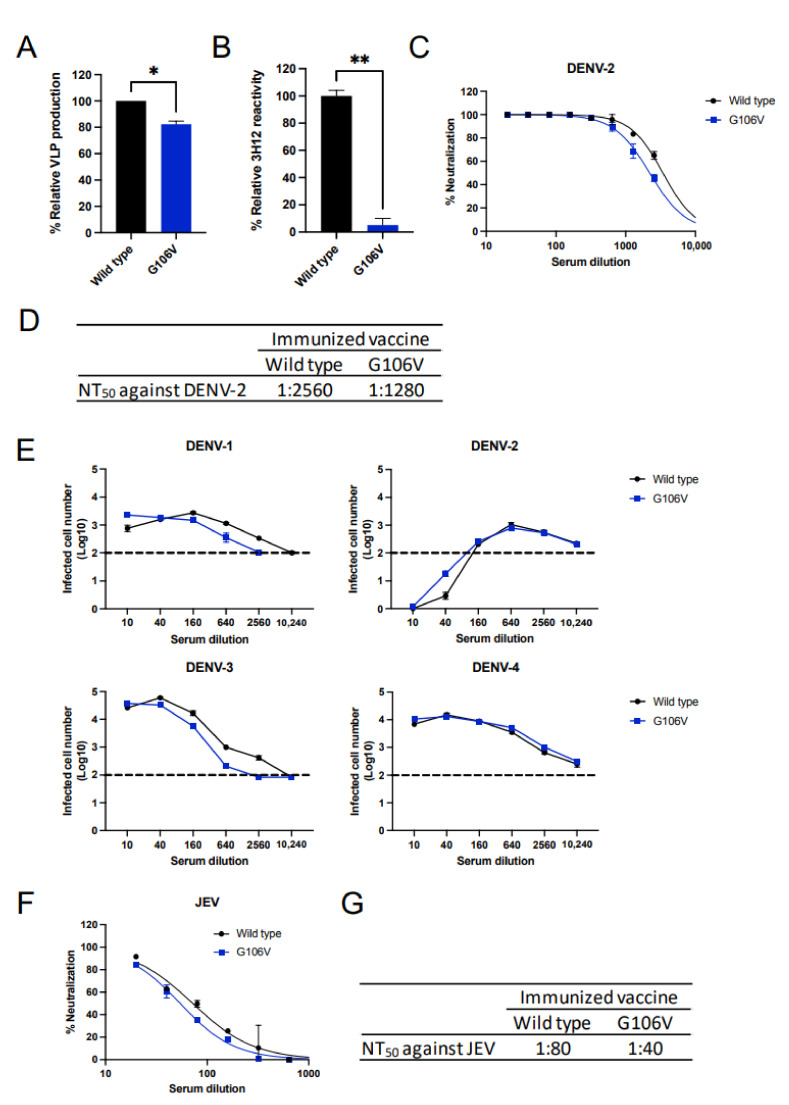
Effect of G106V mutation on dengue DNA vaccine. (**A**) Relative VLP production in dengue DNA vaccine-transfected cells. VLPs were produced in 293T cells transfected with DNA vaccines, which were quantified using ELISA. The VLP production was expressed as the OD of the mutant vaccine relative to that of the wild-type vaccine (OD of mutated dengue vaccine/OD of wild-type vaccine). (**B**) Reactivity of 3H12 to FL-mutated DENV VLPs. The VLPs were produced in cells transfected with mutated DNA vaccines. The reactivity of 3H12 was expressed as the OD of the mutated VLP relative to that of the wild-type VLP (OD of mutated VLP/OD of wild-type VLP). The Student’s *t*-test was performed to analyze the statistical significance of the comparison between the wild-type and mutant vaccines. Statistically significant values (* *p* < 0.05, ** *p* < 0.01) are represented in the graph. (**C**) Neutralizing activity against DENV-2. (**D**) NT_50_ values against DENV-2. NT_50_ was calculated as the maximum serum dilution yielding >50% neutralization. (**E**) ADE activity against four serotypes of DENV. Dotted lines indicate the baseline of infected cells in the control (100 infected cells; log_10_ 2.0). Complement was not added in this assay. (**F**) Neutralizing activity against JEV. (G) NT_50_ values against JEV. NT_50_ is calculated as a maximum serum dilution yielding >50% neutralization.

Due to the 106th amino acid residue being reportedly involved in epitopes within the FL region of DENV [47], we hypothesized that the G106V mutation reduces the induction of ADE-prone antibodies even in the dengue vaccine model. Thus, we introduced the G106V mutation to the plasmid encoding the prM–E of DENV-2 (dengue DNA vaccines). The G106V mutation led to a slight reduction in VLP production (Figure 6A). However, the VLPs produced by G106V completely lost reactivity to 3H12 (Figure 6B). These data confirmed that the dengue DNA vaccine with the G106V mutation is promising in reducing the induction of ADE-prone antibodies.

The dengue DNA vaccines were administered thrice at 2-week intervals. The mutations did not compromise the induction of neutralizing antibodies against DENV-2; the difference was two-fold (Figure 6C,D). However, unlike the JE vaccine, the mutation did not reduce the induction of cross-reactive antibodies against DENV-4, as evaluated using the ADE assay (Figure 6E). The ADE activity against DENV-1 and DENV-3 was approximately two-fold lower, which was not as high as the reduction in ADE activity in the JE vaccine. Moreover, the difference in neutralizing titers against JEV was two-fold between the wild-type and G106V dengue DNA vaccines (Figure 6F,G). Altogether, G106V does not reduce the induction of cross-reactive antibodies against DENVs and JEV in the dengue DNA vaccine model.

## 4. Discussion

Flavivirus infection is an important health problem worldwide. As a specific antiviral treatment against flavivirus infection is not yet available, vaccination is the universal prevention method. Considering that JE vaccination possibly causes the ADE of DENV infections, it is necessary to eliminate the potential risk of ADE through vaccination. In this study, we demonstrated that FL mutations, particularly G106V, reduced the induction of cross-reactive ADE-prone antibodies in the JE vaccine. Furthermore, this mutation did not compromise the neutralization and protection efficacy of the vaccine.

Epitope modification is a promising approach for reducing the induction of ADE-prone antibodies. Regarding FL-modified flavivirus vaccines, other studies have shown that G106D and L107D mutations reduce ADE activity in dengue vaccines [30,31]. Furthermore, one study reported that W101R and L107R reduced ADE activity in the Zika vaccine [33]. We had previously reported that L107F mutation reduced ADE activity in dengue DNA vaccines [32]. In comparison with those reports, the present study focused on the JE vaccine. We demonstrated that FL modification remains a promising approach for JE vaccination. Moreover, this is the first study to report the application of G106V mutation in flavivirus vaccines. Further studies on the comparison of G106V mutation with other FL mutations for reducing ADE activity are warranted.

The advantage of a point mutation on epitope modification is that a point mutation does not severely impair antigenic structure or immunogenicity. The G106V mutation in this study did not compromise the induction of neutralizing antibodies and protection efficacy in the mouse lethal challenge experiment (Figure 5D,E). This could be attributed to the fact that antibodies targeting the FL region are generally weakly neutralizing [22]. This warrants the potential application of the G106V mutation in JE vaccines.

As we investigated the effect of mutations using a DNA vaccine model, the application of G106V mutation in other vaccine platforms is an important topic because most of the currently available JE vaccines are inactivated or live attenuated [2]. This difference in vaccine platforms may cause a difference in the induced antibody profile. In this study, the G106V mutation was introduced into the escape mutant (Figure 1A), which indicates that the virus with the G106V mutation is still replicative, and the mutation does not severely impair viral replication. Therefore, JEV harboring G106V could be used for live attenuated and inactivated vaccines. The application of G106V mutation to these vaccine platforms using reverse genetics system is necessary to confirm whether ADE induction is reduced even in these vaccines [48].

Glycine at the 106th amino acid position on the E protein is highly conserved among mosquito-borne flaviviruses, including JEV and DENV as well as the West Nile virus, Zika virus, and yellow fever virus. In terms of reductions in ADE activity, G106V is superior to L107F in the JE vaccine, and the G106D mutation reportedly reduced the induction of ADE-prone antibodies in the dengue vaccine model [30,31]. Therefore, the G106V mutation could be applied to vaccines against other flaviviruses. However, when the G106V mutation was introduced into the dengue DNA vaccine in this study, it did not reduce ADE activity (Figure 6). This could be attributed to the difference in the 3H12 epitope between DENV and JEV. The escape mutant generated using DENV-1 and 3H12 did not acquire the G106V mutation, implying that G106V is not as critical as L107F in terms of the 3H12 epitope in DENV [32]. Another possible reason is the presence of cross-reactive ADE-prone epitopes other than the FL region among the four serotypes of DENV [47]. It is essential to consider the introduction of mutations other than the FL region to reduce ADE activity in dengue vaccines. Other studies, as well as our previous study, introduced mutations outside of the FL region, including N8R, D87N, K310D/E, E311K/R, and P394Q/R, and confirmed a reduction in ADE activity [30,32,49]. Studies exploring the combination of these mutations with G106V would be of significant interest.

Our previous study demonstrated that L107F suppressed ADE activity in all serotypes of the dengue vaccine [32]. This was a subclass-dependent reduction in ADE activity through the induction of Th1-biased immunity and complement-dependent neutralization. Meanwhile, the G106V mutation in this study reduced ADE activity in the presence or absence of the complement, indicating a subclass-independent reduction in ADE activity. Although these two residues are adjacent, L107F and G106V functioned differently with regards to their reduction of ADE activity.

A limitation of this study is that we examined reductions in ADE activity via an in vitro ADE assay using K562 cells. Therefore, an in vivo ADE model must be used to determine whether G106V mutation reduces ADE activity [31,49]. Furthermore, we used a mouse model for vaccine immunization. The antibody profile induced by flavivirus infection varies between mice and humans [50]. Additionally, the IgG subtype’s and complement’s influence on ADE activity varies between mice and humans. Therefore, further studies using other animal models are needed to confirm the universality of G106V mutation in reducing ADE activity.

## 5. Conclusions

We identified a novel epitope residue in the E protein of JEV, G106V, which is recognized by an ADE-prone antibody. By introducing this mutation, we successfully developed JE vaccines that reduced the induction of cross-reactive ADE-prone antibodies against DENV. The G106V mutation is applicable to JEV irrespective of the strain, but not to DENV-2. Further studies exploring the combination of G106V with other FL mutations, the application of G106V mutation to other vaccine platforms, and performing in vivo ADE assays must be considered.

## Data Availability

All data are available in the main text or Supplementary materials.

## Supplementary Materials

The following supporting information can be downloaded at: https://www.mdpi.com/article/10.3390/vaccines10091411/s1, Table S1: Primers used in this study, Figure S1: Determination of 50% lethal dose. A serially diluted JEV P3 strain was inoculated intraperitoneally into six or seven 6-week-old BALB/c mice to determine the 50% lethal dose (LD_50_).
