# Supplementary material for: Japanese Encephalitis DNA Vaccines with Epitope Modification Reduce the Induction of Cross-Reactive Antibodies against Dengue Virus and Antibody-Dependent Enhancement of Dengue Virus Infection
Source: Vaccines (Basel). 2022 Aug 28;10(9):1411. doi: 10.3390/vaccines10091411 (PMC9506301; doi:10.3390/vaccines10091411)
Supplement: Supplementary file 1 [file vaccines-10-01411-s001.zip › vaccines-1834888-supplementary.pdf]

Table S1. Primers used in this study.

Description	Direction	Sequence
JEV Nakayama strain prM-E cloning into pcDNA3.1	Forward	5′-CCG<u>GAATTC</u>ACCATGGAAGGCTCAATCATGTGG-3′
	Reverse	5′-GGC<u>CTCGAG</u>TTAAGCATGCACATTGGTCGC-3′
JEV Beijing-1 strain prM-E cloning into pcDNA3.1	Forward	5′-CCG<u>GAATTC</u>ACCATGGAAGGCTCAATCATGTGG-3′
	Reverse	5′-GCT<u>ACCAATG</u>TGCATGCTTA-ACTCGAGGCC-3′
JEV P3 strain prM-E cloning into pcDNA3.1	Forward	5′-CCG<u>GAATTC</u>ACCATGGAAGGCTCAATCATGTGG-3′
	Reverse	5′-GCG<u>ACCAATG</u>TGCATGCTTA-ACTCGAGGCC-3′
DENV-2 NGC strain prM-E cloning into pcDNA3.1	Forward	5′-CGG<u>GATATC</u>ACCATGAACAGGAGACGCAGAACT-3′
	Reverse	5′-GGC<u>CTCGAG</u>TTAGGCCTGCACCATAACTCC-3′
Sequence primer designed on CMV promoter	Forward	5′-GGCGTGTACGGTGGGAGGTC-3′
JEV prM-E sequence primer 1	Forward	5′-AGGCATTCCAAGACTAGTAGGAGATCCGTG-3′
JEV prM-E sequence primer 2	Forward	5′-GGGTCACAGGAAGGAGGC-3′
JEV prM-E sequence primer 3	Reverse	5′-AGTCGTTGAAAAGGCTTTGCC-3′
DENV-2 prM-E sequence primer 1	Forward	5′-GCATAGGAATATCAAATAGAG-3′
DENV-2 prM-E sequence primer 2	Forward	5′-GCTCTCCGAGAACGGGCCTCG-3′
DENV-2 prM-E sequence primer 3	Forward	5′-AACCTCCATTCGGAGACAGC-3′
JEV Nakayama G106V introduction	Forward	5′-GTACTTTTCGGGAAGGGAAGCATTGAC-3′
	Reverse	5′-ACATCCGTTGCCCCACCCACGATCAG-3′
JEV Nakayama L107F introduction	Forward	5′-GGATTTTTCGGGAAGGGAAGCATTGAC-3′
	Reverse	5′-ACATCCGTTGCCCCACCCACGATCAG-3′
JEV Nakayama G106V/L107F introduction	Forward	5′-GTATTTTTCGGGAAGGGAAGCATTGAC-3′
	Reverse	5′-ACATCCGTTGCCCCACCCACGATCAG-3′
JEV Beijing-1 G106V introduction	Forward	5′-GTACTTTTCGGGAAGGGAAGTATTG-3′
	Reverse	5′-GATCGTGGGTGGGGCAACGGATGT-3′
JEV P3 G106V introduction	Forward	5′-GTACTTTTCGGGAAAGGAAGCATTG-3′
	Reverse	5′-GATCGTGGGTGGGGCAACGGATGT-3′
DENV-2 G106V introduction	Forward	5′-GTATTATTTGGAAAAGGAGGCATTGTG-3′
	Reverse	5′-ACATCCATTTCCCCATCCTCTGTCC-3′

Underline: Restriction enzyme site.

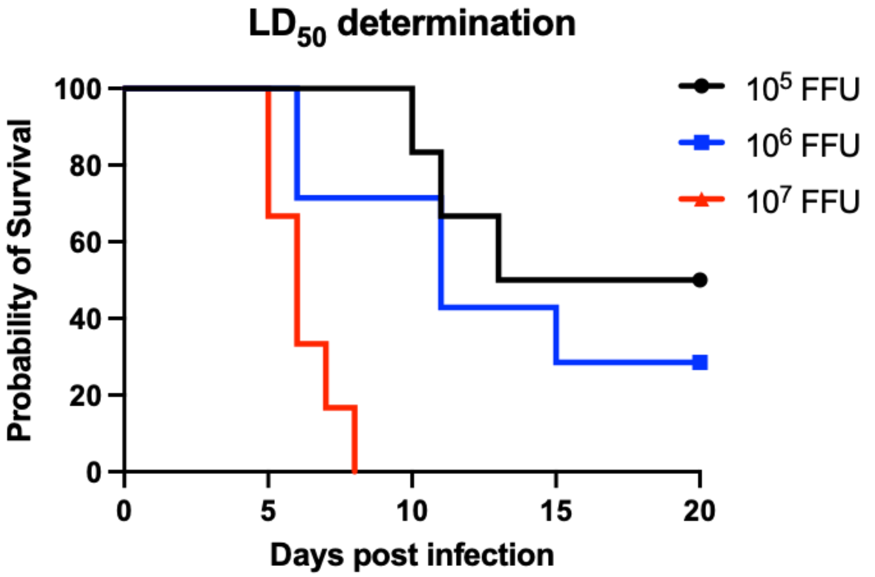

Figure S1. Determination of 50% lethal dose. A serially diluted JEV P3 strain was inoculated intraperitoneally into six or seven 6-week-old BALB/c mice to determine the 50% lethal dose (LD_{50}). The mice were monitored for 20 days. Mice were humanely euthanized if they showed apparent symptoms or a decrease of >20% in their initial body weight. The estimated LD_{50} was 1.4×10^5 focus forming unit (FFU).
